# The parental reflective functioning questionnaire: Development and preliminary validation

**DOI:** 10.1371/journal.pone.0176218

**Published:** 2017-05-04

**Authors:** Patrick Luyten, Linda C. Mayes, Liesbet Nijssens, Peter Fonagy

**Affiliations:** 1 Faculty of Psychology and Educational Sciences, KU Leuven, Leuven, Belgium; 2 Research Department of Clinical, Education and Health Psychology, UCL, London, United Kingdom; 3 Yale Child Study Center, Yale University School of Medicine, New Haven, Connecticut, United States of America; University of New South Wales, AUSTRALIA

## Abstract

This paper reports on three studies on the development and validation of the Parental Reflective Functioning Questionnaire (PRFQ), a brief, multidimensional self-report measure that assesses parental reflective functioning or mentalizing, that is, the capacity to treat the infant as a psychological agent. Study 1 investigated the factor structure, reliability, and relationships of the PRFQ with demographic features, symptomatic distress, attachment dimensions, and emotional availability in a socially diverse sample of 299 mothers of a child aged 0–3. In Study 2, the factorial invariance of the PRFQ in mothers and fathers was investigated in a sample of 153 first-time parents, and relationships with demographic features, symptomatic distress, attachment dimensions, and parenting stress were investigated. Study 3 investigated the relationship between the PRFQ and infant attachment classification as assessed with the Strange Situation Procedure (SSP) in a sample of 136 community mothers and their infants. Exploratory and confirmatory factor analyses suggested three theoretically consistent factors assessing pre-mentalizing modes, certainty about the mental states of the infant, and interest and curiosity in the mental states of the infant. These factors were generally related in theoretically expected ways to parental attachment dimensions, emotional availability, parenting stress, and infant attachment status in the SSP. Yet, at the same time, more research on the PRFQ is needed to further establish its reliability and validity.

## Introduction

We report on the development and initial validation of the Parental Reflective Functioning Questionnaire (PRFQ), a brief self-report measure designed to assess parental reflective functioning (PRF), that is, the capacity to treat the infant as a psychological agent [1, 2].

We first review theoretical assumptions and empirical findings concerning the role of PRF in socioemotional development and the intergenerational transmission of attachment specifically. This is followed by a discussion of the advantages and disadvantages of extant interview and observational measures of (parental) reflective functioning. It is argued that, despite the advantages of these measures, there is a need for a brief self-report measure of PRF that can be used in large-scale studies. In response, we describe the development of the PRFQ and three studies addressing its reliability and validity.

### Parental reflective functioning, attachment, and child development

Reflective functioning, or mentalizing, refers to an individual’s ability “to hold others’ minds in mind” [3–5]. This capacity allows individuals to perceive both the self and others in terms of mental states, thereby making them meaningful, understandable, and predictable. The capacity for reflective functioning is therefore key in our ability to navigate the social world [5]. Unsurprisingly, therefore, impairments in mentalizing, and particularly an absent or compromised capacity to understand oneself and others in terms of mental states, have been shown to play an important role in the development of various psychiatric disorders, most specifically borderline personality disorder [6–11], antisocial personality disorder [12], eating disorders [13–17], and depression [18–20]. Over the past decades, a number of prevention and treatment programs for a variety of disorders and problem behaviors have been developed based on these ideas [6, 21–23] and have been evaluated in randomized controlled trials [24–26].

Parallel to the burgeoning research on the role of reflective functioning in psychopathology, there is increasing interest in the origins of this capacity [27–31]. Studies suggest that mentalizing capacities develop in the context of early secure attachment relationships and foster affect regulation, self-control, and secure attachment in offspring [2, 28]. This has led to a growing attention in both research and clinical practice for the concept of Parental Reflective Functioning (PRF), that is, the caregiver’s capacity to reflect upon his/her own internal mental experiences as well as those of the child, is thought to play a key role in this context [1, 28, 32]. PRF is thought of as a relationship-specific manifestation of the more general capacity for reflective functioning. Although general reflective functioning and parental reflective functioning can be expected to be correlated, these two capacities are not identical. In line with this assumption, a study by Steele et al. [33] reported a correlation of *r* = .50 between general reflective functioning as scored on the Adult Attachment Interview (AAI) [34] and parental reflective functioning as scored on the Parent Development Interview (PDI) [35].

PRF is thought to foster the infant’s own capacity for reflective functioning, which in turn is assumed to play a crucial role in emotion regulation, the development of a sense of personal agency, and secure attachment (i.e., “secure base” feelings that are the basis for a desire to explore the world) [1, 32]. The development of secure attachment and mentalizing is thus thought to depend on the quality of attachment relationships, caregivers’ emotional availability, and the extent to which the infant’s subjective experiences are adequately mirrored by a trusted other, leading to the development of affect regulative processes and self-control (including attention mechanisms and effortful control) as well as the capacity for mentalizing. It is thus not secure attachment or emotional availability per se, but a socializing context that focuses upon mental states, that is thought to foster the development of secure attachment and reflective functioning in infants [2, 36]. This has led to the hypothesis of a so-called “loose coupling” among attachment, emotional availability, and PRF, in that parental secure attachment and high levels of emotional availability are not necessarily expected to be associated with high levels of PRF, although generally they can be expected to be positively related [28]. In contrast, while it is possible that insecurely attached caregivers can have high levels of PRF, it is considerably less likely compared to securely attached caregivers. Indeed, disruptions in early attachment relationships and later trauma are assumed to have the potential to impair both the capacity for mentalizing and the development of a coherent self-structure, leading to the loss of mentalizing in emotionally intense relationship contexts, and the re-emergence at such stressful times of modes of thinking about subjective experiences that antedate full mentalizing [37]. In the context of PRF, these pre-mentalizing modes of experiencing subjectivity on the part of caregivers are typically expressed as a tendency to be overly certain about the mental states of their child, often associated with making malevolent attributions and an inability to enter into the child’s internal subjective world, features that studies suggest are characteristic of many parents with severe mentalizing problems [32, 38–40]. Hence, whereas recognizing the opacity of mental states and showing a genuine interest and curiosity in the internal world of the child are considered to be the hallmarks of genuine parental reflective functioning [1], insecurely attached parents seem to lack both of these capacities.

Congruent with these assumptions, a number of studies have found that the parents’ general capacity for reflective functioning is associated with the development of secure attachment and mentalizing in their children [41, 42]. Other studies reported that found that low levels of PRF specifically were associated with disrupted maternal behavior and infant insecure attachment [43, 44]. Similarly, Meins et al. [45] found that maternal mind-mindedness (MMM), a concept that is closely related to PRF [28], prospectively predicted child attachment as assessed with the Strange Situation Procedure (SSP) [45–48], social-cognitive performance in children at 55 months [49], and effortful control at 18 and 26 months [50]. Moreover, greater parental mind-mindedness was associated with prenatal autonomous parental AAI classification, higher reflective functioning, and infant—parent attachment security [30]. However, these associations were found only for appropriate, and not for inappropriate, nonattuned, mind-related comments. This raises the issue of “parental hypermentalizing” or “pseudomentalizing”, a type of mentalizing that is often highly cognitive, overly elaborate, and may be experienced by the child as intrusive, leading to a misinterpretation or “misreading” of the child’s mind [5]. Disruptions in PRF thus may be either *deficient*, expressed in limited, concrete, and stimulus-bound reflective functioning, or *excessive*, as is shown in parental accounts of their child’s behaviors and attitudes that go far beyond the data, that are characterized by undue certainty about the mental states of the child, and that are often distorted and intrusive, and sometimes paranoid [18, 51, 52].

### Assessment of (parental) reflective functioning

There are currently many measures to assess (parental) reflective functioning [5]. A recent review of measures of PRF in the first three years of life by Schiborr et al. [53], identified at least 15 separate measurement approaches to assessing maternal mentalizing in this age period alone. These include assessments of maternal narratives for reflective functioning [1, 54], insightfulness [55, 56], meta-emotional representation [57], and the proclivity to attribute meaning to the child, so-called Maternal Mind-Mindedness (MMM) [58], and observational tools to assess the mother’s treatment of the infant as a mental agent capable of intentional action [45], the use of mental-state terms [59, 60], mental-state language [61], and mental-state references [62] in relation to the child.

These measures have many advantages, particularly in that they often yield clinically rich and detailed data that allow an in-depth exploration of the role of (parental) reflective functioning in child development that is impossible to achieve with simple self-report questionnaires. However, their time- and cost-intensive nature hampers their use in large-scale studies [5].

### The present study

This paper introduces the PRFQ, a brief, multidimensional assessment of PRF that is easy to administer to parents with a wide range of socioeconomic and educational backgrounds. Hence, care has been taken to restrict the number of items, and to formulate short and easy-to-understand items that can either be completed by parents on their own or be read to them (which may be more appropriate in vulnerable samples). Because of the current interest in the role of PRF in the intergenerational transmission of attachment in early childhood, the PRFQ was primarily designed for parents with children 0–5 years of age. Particularly in the early stages of development, when communication between parent and infant is mainly nonverbal, the ability to be sensitive and responsive to the infant’s emotional cues may be an important determinant of the subsequent socioemotional development of the child [43]. In this context, it is often assumed that high levels of PRF are expressed in active interest and curiosity in mental states leading to a search for understanding, which is considered by some to be the hallmark of healthy PRF, together with an acknowledgment of the opacity of mental states [63]. From age 3 onward, but particularly after age 5, because of children’s progress in terms of language development and understanding of the social world, the process of parental mentalizing becomes largely based on internal features of the child (the child’s wishes, desires, fears, etc.), which might in part entail different processes and capacities [5]. Yet, ultimately, it is an empirical question whether the PRFQ is equally valid for children in this age range, and whether it may also be used in parents with children above the age of 5.

Based on the theoretical formulation concerning PRF reviewed above, we formulated items that aimed to capture three key features of PRF [1, 33, 63]: (a) interest and curiosity in mental states, (b) the ability to recognize the opacity of mental states, and (c) nonmentalizing modes characteristic of parents with (severe) impairments in PRF (e.g., malevolent attributions, inability to enter into the subjective world of the child), given that mentalization-based approaches emphasize the centrality of these modes of thinking, which antedate full mentalizing, in understanding the links between impairments in mentalizing, insecure attachment, and psychopathology [4, 7].

The present paper presents three studies in a series investigating the psychometric features of the PRFQ. Other studies have reported expected relationships between the PRFQ and tolerance of infant distress in a simulated baby paradigm [64, 65], and with neural correlates of infant face and cry perception using event-related potentials (ERPs) [66]. The aims, design, and methods of each of the three present studies are discussed in detail in the following sections.

## Study 1

Study 1 presents the initial development and validation of the PRFQ in a sample of mothers with infants aged 0–3 years (*N* = 299). Because the PRFQ was designed for use in both high- and low-functioning samples, this sample was heterogeneous with respect to infant age, maternal socioeconomic background, and risk status. The aims of this study were threefold. First, we wanted to investigate the factor structure of the PRFQ using both exploratory principal component analysis (PCA) and confirmatory factor analysis (CFA). Based on the theoretical principles guiding our creation of the questionnaire items, we expected to find a three-factor structure, with items capturing three key dimensions of PRF, that is, (a) interest and curiosity, (b) recognizing the opacity of mental states, and (c) pre-mentalizing modes of reflecting about the infant’s mental states. Second, this study was also designed to explore the relationship between the PRFQ, demographic features (e.g., maternal age, level of education, duration of partner relationship), and symptomatic distress, as these features may influence PRF. For instance, it could be argued that PRF increases with maternal age [67]. Similarly, higher levels of education may be positively related to PRF [68]. Conversely, symptomatic distress may impair reflective functioning [69]. Third, we expected to find theoretically meaningful correlations between the PRFQ, adult attachment dimensions, and emotional availability. Based on the literature reviewed above, positive features of PRF (e.g., interest and curiosity) should be correlated negatively with attachment anxiety and avoidance, and positively with features of maternal emotional availability. Furthermore, it can be expected that high levels of maternal reflective functioning would be associated with higher levels of emotional availability in the child, leading to a virtuous cycle characterized by greater emotional availability between mother and child [1, 32]. Hence, we also expected positive correlations between positive features of PRF and mother—infant and child emotional availability, and the converse for negative features of PRF.

### Methods

#### Participants and procedures

Mothers in Study 1 were recruited by research assistants in a wide range of childcare centers in Belgium between October 2009 and September 2011 as part of an ongoing longitudinal study of child development. Recruitment included oversampling for women with low socioeconomic status and at-risk status (e.g., by recruiting mothers from state subsidized day-care centers for mothers with low socio-economic and at-risk status) to ensure variability in levels of PRF, attachment, emotional availability, and psychological distress. Inclusion criteria were: (a) sufficient mastery of the Dutch language and (b) being a biological mother of a child aged between 0 and 36 months. Eligible mothers were contacted through day-care services and were invited to participate in a study about parenthood. Demographic data are summarized in Table 1.

**Table 1:** Demographic features of mothers in Study 1.

Parameter	Mothers (*N* = 299)
Age (years)^a^	31.06 (4.51)
Belgian nationality (%)	95.70
Duration of relationship (months)^a^	111.83 (50.74)
Duration of living together (months)^a^	79.99 (38.56)
Duration of marriage (months)^a^	60.35 (40.53)
Educational level (%):	
Primary education	2.30
Secondary education	18.80
Higher education (3 years)	35.60
Higher education (>3 years)	43.30
Work (hours per week)^a^	32.55 (11.93)
Work (days per week)^a^	4.43 (1.37)
Working status (%)	
Unemployed	4.00
Working at home	3.00
Working outside home	93.00

^a^ Mean (*SD*)

Participation was entirely voluntary and full anonymity was guaranteed. When mothers agreed to participate, they were requested to sign an informed consent form and to complete a booklet of questionnaires (see below). A few weeks later, the booklets were collected (in a sealed envelope) at the day-care center. This study was approved by the Ethics Committee of KU Leuven (the University of Leuven), Belgium.

In total, 301 mothers completed questionnaires. One mother was excluded from the study because more than half of the item scores were missing from her questionnaire, and another mother was excluded because her child was older than 36 months. The final sample therefore consisted of 299 mothers. Mothers were on average 31.06 years old (*SD* = 4.51; range 19–44) and most had Belgian nationality (*n* = 286, 95.7%). Information about nationality was missing for two mothers. More than two-thirds of the mothers had a higher educational level (college/university; 78.7%) and were working outside of their home (93.0%). Two hundred and sixty-seven mothers (89.3%) were in a partner relationship, with an average relationship duration of 111.83 months (*SD* = 50.74). The mean age of their infants was 19.03 months (*SD* = 8.87; range 2–36). The gender of the children was roughly equally divided, with 154 boys (51.5%) and 144 girls (48.2%). Two hundred and ninety-two (98.0%) of the infants had Belgian nationality. Information about nationality and gender was missing for one infant.

#### Measures

The development and validation of the PRFQ closely followed guidelines for the development of psychometric tests [70]. In a first stage, items were generated based on a careful screening of the relevant literature on mentalizing and social cognition [4, 6, 28, 29, 71], and PRF in particular [1, 28, 63]. In addition, items were formulated based on descriptions and examples in the reflective functioning manual for the AAI [72], the reflective functioning manual for the PDI [63], and the Reflective Function Rating Scale [73]. We formulated items both positively (i.e., higher scores reflecting higher levels of PRF) and negatively (i.e., higher scores reflecting lower levels of PRF). Moreover, some items were formulated such that both high and low scores reflected low PRF while scores in the middle of the scale reflected higher levels, as high levels of PRF entail acknowledgement of the opaqueness of mental states. Elimination of items that were highly similar resulted in a pool of 57 items.

Next, 20 experts were asked to rate each of the 57 items in terms of prototypicality for a high-mentalizing and a low-mentalizing mother, respectively. The first 10 experts were instructed to rate all 57 items for prototypicality with a high-mentalizing mother in mind, the other 10 experts were asked to rate all items keeping a low-mentalizing mother in mind. Raters were asked to use a partly fixed distribution, such that only eight items could receive the maximum score of 7 (most prototypical) and only 10 items could receive a score of 6 (next most prototypical); for the remainder of the items, experts were free to give any score between 0 (not at all prototypical) and 5 (somewhat prototypical). Hence, we were mainly interested in identifying those items with high prototypicality (i.e., those assigned scores of 6 and 7). In addition, experts had to indicate on a 0–100 scale how well they thought all the items captured the capacity for maternal reflective functioning of a high- or low-mentalizing mother, respectively. Finally, the experts were given the opportunity to provide additional comments and suggestions concerning the items and sorting task.

Results showed that the experts thought that the items captured the capacity for maternal reflective functioning quite well, as indicated by a median score for high-mentalizing mothers of 75 for both low- and high-mentalizing mothers. Moreover, for the high-mentalizing mothers rating task, 17 of a possible 18 items received an average score across experts of between 6 and 7 for prototypicality; for the low-mentalizing mothers task this was the case for 14 of a possible maximum of 18 items. Items that received high scores for prototypicality for high-mentalizing mothers typically received very low scores for prototypicality for low-mentalizing mothers and vice versa. On the basis of these results and additional suggestions from the experts concerning the clarity and comprehensibility of some items, we reformulated several items and removed others because of their similarities, leading to a set of 39 items. (The complete set of items can be obtained from the first author.)

Symptomatic distress was assessed with the Brief Symptom Checklist (Korte Klachtenvragenlijst; KKL) [74]. The KKL is a 14-item self-report questionnaire on which every item corresponds to a group of familiar psychiatric complaints (i.e., depression, anxiety). The KKL has shown good validity and reliability [74]. The estimate of internal consistency in this study was α = .77.

The Experience of Close Relationships—Revised (ECR-R) [75] was used to assess attachment anxiety and avoidance. This scale contains 36 items derived from an item response theory analysis of existing self-report measures of adult attachment, assessing two dimensions underlying adult attachment: attachment avoidance (i.e., discomfort with closeness and discomfort with depending on others 18 items; e.g., “I worry that my romantic partners won’t care about me as much as I care about them”) and attachment anxiety (i.e., fear of rejection and abandonment; 18 items; e.g., “I prefer not to show a partner how I feel deep down”). All items are scored using a 7-point Likert-type scale. Estimates of internal consistency in this study were α = .92 for both attachment avoidance and anxiety.

The Emotional Availability—Self Report (EA-SR) [76, 77] assesses the emotional availability of the parent, the child, and aspects of dyadic attunement. The EA-SR consists of 32 items pertaining to two parental subscales (Intrusiveness [six items; e.g., “I find it hard to see my child playing on his own, I prefer to do things together when we’re at home”] and Hostility [two items; e.g., “It happens that I shout at my child to make something clear”]), one child subscale (Involvement [nine items; e.g., “My child is able to get my attention for his/her play”]), and two dyadic subscales (Mutual Attunement [10 items; e.g., “I succeed in adjusting to my child’s behaviors and actions when necessary”] and Affect Quality [five items; e.g., “My child clearly enjoys being together with me”]). Each scale is rated on a 5-point Likert scale: 0 (disagree completely), 1 (tend to disagree), 2 (neutral), 3 (tend to agree), 4 (agree completely). In previous studies [76, 77], estimates of internal consistency were good (range between α = .71 and .84), except for the affect quality subscale (α = .49). The validity of the EA-SR has been supported by significant and meaningful correlations with corresponding subscales of the Emotional Availability Scales rated on mother—infant interactions, except for the Intrusiveness subscale [77]. In this study, we used the single subscales and also calculated a parental emotional availability scale (by reverse coding the Intrusiveness and Hostility subscales, such that higher scores reflected higher parental emotional availability, and then averaging both scores), a child emotional availability scale (consisting of the Involvement subscale), and a dyadic emotional availability scale (by averaging the scores on the Mutual Attunement and Affect Quality subscales). Estimates of internal consistency in this study were α = .81 for Intrusiveness, .87 for Hostility, .84 for Involvement, .74 for Mutual Attunement, and .60 for Affect Quality. Thus, as in other studies, the internal consistency of the Affect Quality subscale was relatively low and thus findings with this scale need to be interpreted with caution.

#### Data analysis

PCA with varimax and promax rotation was used in a first stage to explore the factor structure of the PRFQ. The following criteria were used to identify the number of factors: (a) the number of components with an eigenvalue >1, (b) the scree test, and (c) interpretability. Next, CFA [78] was used to investigate the fit of the obtained model. The following fit indices were used: the χ^2^/df ratio, the root mean square error of approximation (RMSEA), the comparative fit index (CFI), and the non-normed fit index (NNFI). A model in which χ^2^/df was ≤3, CFI and NNFI were greater than .90, and the RMSEA index was between .00 and .06 with confidence intervals between .00 and .08 was considered acceptable [79].

Relationships between the PRFQ subscales, demographic features, symptomatic distress, attachment, and emotional availability were investigated using Pearson correlation or Spearman rho coefficients, as appropriate. AMOS Version 4.01 [80] was used for CFA using the maximum-likelihood method. All other analyses were done using SPSS 19.0.

### Results

#### Factor structure of the PRFQ

The scree test clearly indicated a three-factor solution. Eigenvalues of the first three factors were 5.48, 3.41, and 3.29, explaining 31.24% of the variance. Solutions with more than three factors were difficult to interpret because of the small number of items comprising the fourth and fifth factors. Results of a PCA on the raw items and on the rescored items (with all items rescored so that higher scores reflected higher levels of PRF) were highly similar. Varimax- and oblimin-rotated solutions of the three factors were also similar.

The content of these three factors was readily interpretable and was closely aligned with theoretical descriptions of key features of PRF. We labeled the first factor Pre-Mentalizing (PM), as items seemed to capture a nonmentalizing stance, and malevolent attributions and an inability to enter the subjective world of the child in particular (e.g., “My child cries around strangers to embarrass me”). The second factor consisted of items related to the recognition of the opacity of mental states. We chose the more descriptive label Certainty about Mental States (CMS), as scores on this scale may range from a tendency of parents to be overly certain about the mental states of their child (i.e., to not recognize the opacity of mental states), reflecting intrusive mentalizing or hypermentalizing, to hypomentalizing, that is, an almost complete lack of certainty about the child’s mental states. The third factor captured items reflecting Interest and Curiosity (IC) in mental states, a key feature of PRF (e.g., “I like to think about the reasons behind the way my child behaves and feels”). Again, whereas low levels might reflect an absence of interest in one’s infant’s mental states, very high scores might reflect intrusive hypermentalizing.

**Fig 1:**
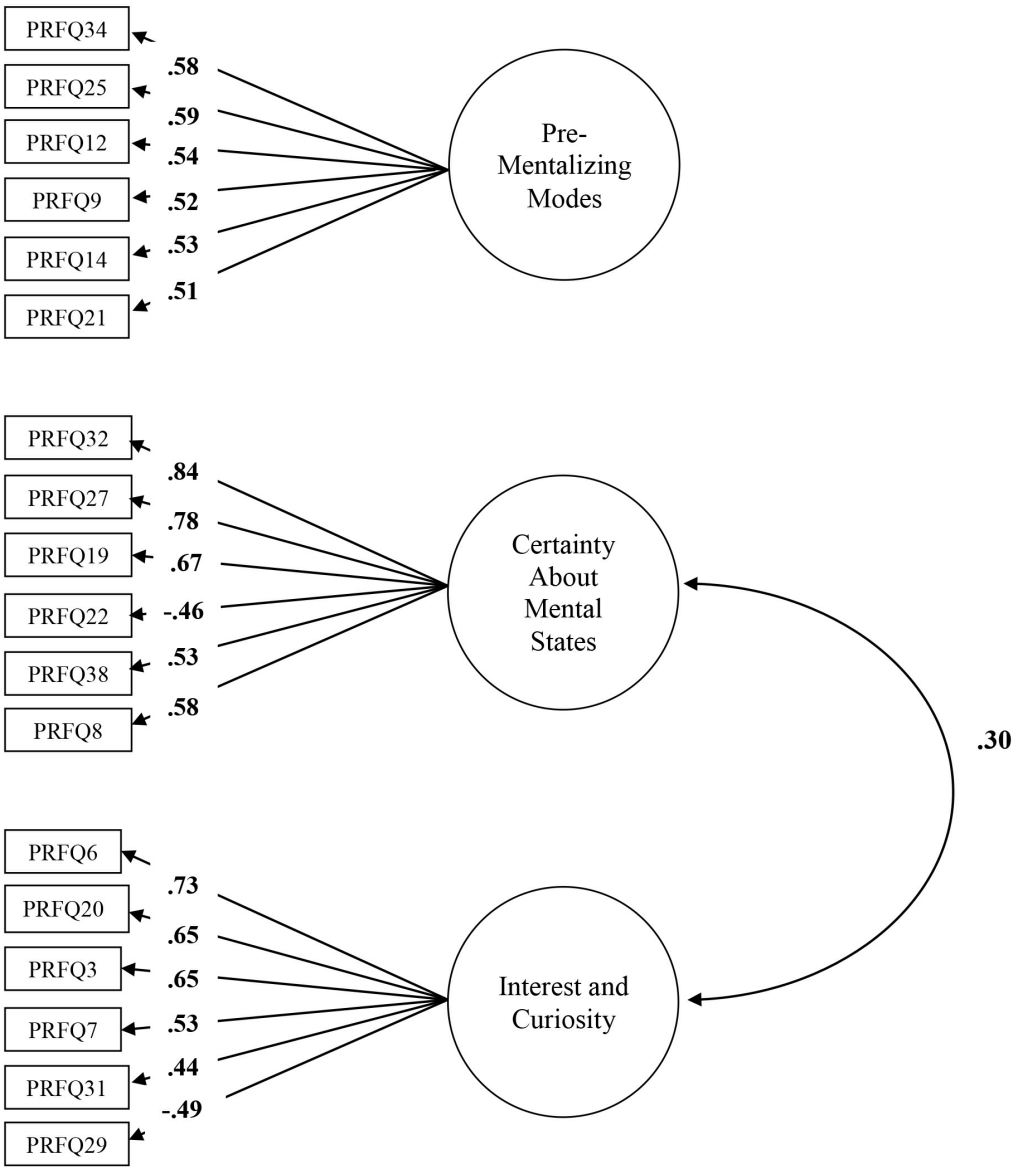
CFA in Study 1. Residuals and correlations between residuals are omitted for clarity of presentation. Rectangles indicate measured variables and circles represent latent constructs. Standardized maximum likelihood parameters are used. Bold estimates are statistically significant.

Next, we ran a CFA model with these three factors as latent factors. Because our aim was to develop a brief measure of PRF, we selected the six items that had the highest loading on their respective factor in the PCA. This three-factor solution had a relatively good fit: χ^2^ = 384.44, df = 132, *p* < .001, χ^2^/df = 2.64; RMSEA = .07 (95% confidence interval [CI] [.07, .08]); CFI = .84, NNFI = .81. Modification indices suggested to add error covariances between several items, which resulted in a model with a good fit: χ^2^ = 217.73, df = 123, *p* < .001; χ^2^/df = 1.77; RMSEA = .05 (CI [.04, .06]); CFI = .91, NNFI = .91. All items had substantial and significant loadings in the expected direction on their respective factors. Fig 1 presents the final model. PM did not correlate with the other two factors. However, CMS and IC were significantly correlated (*r* = .30, *p* < .01). Next, we calculated subscales based on the final model. Estimates of internal consistency (Cronbach’s alpha) were .70, .82, and .75 for PM, CMS, and IC, respectively.

#### Relationships with demographic features

Most correlations with demographic features were non-significant or were very small (Table 2), with the exception of PM. PM was negatively correlated with parental age (*r* = -.16, *p* < .01), parental level of education (*r* = -.42, *p* < .001), working hours (*r* = -.31, *p* < .001), and working days (*r* = -38, *p* < .001). Moreover, there were slight negative correlations between IC and the duration of the partner relationship (*r* = -.15, *p* < .05) and the time the parents had been living together (*r* = -.24, *p* < .01). Given that there may be curvilinear relationships, particularly for CMS and IC, we investigated this possibility, but no significant curvilinear relationships were found.

**Table 2:** Correlations among the PRFQ subscales and demographic features in Study 1.

	Age of Parent	Parent Level of Education	Working Hours	Working Days	Duration of Relationship	Duration of Living Together	Age of Child
Pre-Mentalizing Modes	-.16**	-.42**	-.31**	-.38**	.01	.07	.03
Certainty about Mental States	-.01	-.01	-.01	-.01	-.01	-.03	.07
Interest and Curiosity	-.08	.05	.05	.13*	-.15*	-.24**	-.08

* *p* < .05 (two-tailed),

** *p* < .01 (two-tailed).

#### Relationships with symptomatic distress and attachment

Contrary to expectations, CMS and IC were relatively independent of symptoms (*r* = -.16, *p* < .01 and *r* = .11, *p* = .06, respectively), attachment avoidance (*r* = -.14, *p* < .05 and *r* = -.10, *ns*, respectively) and attachment anxiety (*r* = -.12, *p* < .05 and *r* = .03, *ns*, respectively; Table 3). In contrast, as expected, PM was positively correlated with symptoms (*r* = .29, *p* < .001) and highly positively correlated with both attachment anxiety (*r* = .49, *p* < .001) and attachment avoidance (*r* = .49, *p* < .001; see also Table 3).

**Table 3:** Correlations among the PRFQ subscales, symptomatic distress, and maternal attachment in Study 1.

PRFQ subscales	Attachment Avoidance	Attachment Anxiety	Symptomatic Distress
Pre-Mentalizing Modes	.49**	.49**	.29**
Certainty about Mental States	-.14*	-.12*	-.16*
Interest and Curiosity	-.10	.03	.11

* *p* < .05 (2-tailed),

** *p* < .01 (2-tailed).

#### Relationships with emotional availability

As expected, PM was negatively correlated with self-reported emotional availability of the parent (*r* = -.15, *p* < .05), the child (*r* = -.30, *p* < .001) and particularly the dyad (*r* = -.59, *p* < .001). The pattern for CMS and IC was more differentiated. CMS was, in line with expectations, positively correlated with parent (*r* = .19, *p* < .01) and dyadic (*r* = .36, *p* < .001) emotional availability, but not with child emotional availability, while IC was slightly negatively correlated with parent emotional availability (*r* = -.15, *p* < .01), but positively correlated with child emotional availability (*r* = .22, *p* < .01).

Looking at the subscales in detail, PM was, as expected, highly negatively correlated with all emotional availability dimensions, that is, mutual attunement (*r* = -.52, *p* < .001), child involvement (*r* = -.30, *p* < .001), and affect quality (*r* = -50, *p* < .001), and was positively correlated with perceived hostility (*r* = .25, *p* < .001) (Table 4), with one exception: PM was not related to perceived intrusiveness (*r* = -.10, *ns*).

**Table 4:** Correlations among the PRFQ subscales and emotional availability in Study 1.

	Mutual Attunement	Child Involvement	Affect Quality	Intrusiveness	Hostility	Parent Emotional Availability	Child Emotional Availability	Dyad Emotional Availability
Pre-Mentalizing Modes	-.52**	-.30**	-.50**	-.10	.25**	-.15*	-.30**	-.59**
Certainty about Mental States	.38**	.09	.16**	.20**	-.35**	.19**	.09	.36**
Interest and Curiosity	.07	.22**	.06	.33**	-.05	-.15**	.22**	.07

* *p* < .05 (2-tailed),

** *p* < .01 (2-tailed).

CMS was positively correlated with both affect attunement (*r* = .38, *p* < .001) and affect quality (*r* = .16, *p* < .01), was not related to child involvement (*r* = .10, *ns*), negatively related to hostility (*r* = -.35, *p* < .01), but slightly positively correlated with intrusiveness (*r* = .20, *p* < .01). Finally, IC was positively correlated with perceived child involvement (*r* = .22, *p* < .01) and, like CMS, with intrusiveness (*r* = .33, *p* < .01), and unrelated to the other emotional availability scales.

### Discussion

Both exploratory factor analysis and CFA suggested that three theoretically meaningful factors assessing pre-mentalizing modes, certainty about mental states, and interest and curiosity in mental states underlie the PRFQ items. The CMS and IC subscales were relatively independent of demographic features, symptoms, and attachment avoidance and anxiety. In contrast, PM was negatively correlated with level of education and indices of working status, positively correlated with symptomatic distress, and highly positively correlated with both attachment anxiety and attachment avoidance. These findings are congruent with what has been described as the “loose coupling” between PRF and parental attachment, in that indices of more adaptive PRF seem to be relatively unrelated to attachment, suggesting that these may be two independent factors, at least in this study [4]. These factors were also relatively independent of distress in this study. By contrast, PM, as an indicator of maladaptive mentalizing, was related to demographic features such as level of education and time spent working, symptomatic distress, and insecure attachment. The negative correlation of PM with level of education, working days and working hours, is consistent with theories arguing that individuals that are socioeconomically disadvantaged are also disadvantaged in terms of social learning and thus mentalizing, resulting in a tendency to see others and the world as malevolent [68]. The findings from Study 1 are also congruent with studies suggesting that both distress and the use of attachment hyperactivating and deactivating strategies (associated with attachment anxiety and avoidance, respectively) are related to impairments in reflective functioning, and, in turn, give rise to poor mentalizing [81].

Findings concerning emotional availability shed further light on the features of the different PRFQ subscales. Although infants with more sensitive mothers are more likely to develop secure attachments to their mothers, this relationship is not particularly strong [82]. Findings of this study similarly suggest there is no one-to-one relationship between emotional availability, an index of parental sensitivity, and PRF. The PM subscale, which assesses distortions in mentalizing, seems to be most closely related to dimensions of emotional availability. PM was highly negatively associated with all dimensions of emotional availability, with one important exception: PM was not related to maternal intrusiveness. However, studies have shown that the intrusiveness subscale of the EA-SR scale does not correlate with observer-rated intrusiveness, while the other EAS-SR are significantly correlated with their respective observer-rated scales [77]. It may be that parents show a bias in reporting intrusiveness. Thus, the absence of an association between PM and maternal intrusiveness might reflect reporting bias of mothers with high levels of PM. Further research is needed to investigate this assumption.

Both CMS and IC were generally positively associated with features of emotional availability, but these associations were more modest compared with those with PM. Moreover, CMS and IC were also slightly positively correlated with intrusiveness, suggesting that high levels of CMS and IC are maladaptive in that they may be associated with intrusiveness generally and intrusive hypermentalizing in particular (i.e., assuming that one “knows” everything about one’s infant’s mental states). Again, further research is needed to test these assumptions, and we will return to this issue in Study 3.

Further limitations of this study are that all measures used in the study were based on self-report questionnaires. Despite the fact that the measures included in this study have been validated against observer-rated and interview-based measures, findings in this study might still reflect in part shared method variance. In addition, although we purposefully oversampled mothers with lower socioeconomic and educational status, and the sample included mothers who were recruited through state-subsidized child day-care centers for at-risk mothers, replication of findings in more vulnerable samples is needed. Finally, this study included only mothers, and thus replication of findings in fathers is needed. This is one of the aims of Study 2.

## Study 2

The main aim of Study 2 was to replicate findings from Study 1 and to investigate the factorial invariance of the PRFQ across mothers and fathers. To date, the literature on PRF has almost exclusively focused on mothers. However, fathers’ PRF capacities have also been associated with socioemotional development in their children [28]. Hence, it is important to determine whether the factor structure and correlates of the PRFQ are similar or different in fathers and mothers. Indeed, attachment research, for instance, has reported similar distributions of attachment classifications in mothers and fathers [83]. Likewise, the proportion of mother-infant and father-infant secure attachment categorizations based on the SSP has been found to be similar [83, 84]. A recent meta-analysis found a similar effect size of the association between mother and father attachment representations and secure parent-child attachment [85]. Yet, studies also suggest that many children report a secure attachment with only one parent [84], and that secure attachment to the father may have a protective effect for children with insecure relationships with their mothers [86]. Similarly, PRF in mothers and fathers may have a different impact on the socioemotional development of children, but there is a dearth of studies that have investigated this assumption [30, 41, 87]. Hence, we exploratively investigated potential differences between mothers and fathers in the relationship between the PRFQ and demographic characteristics, symptomatic distress, adult attachment dimensions, and parenting stress. Data for this study were taken from a convenience sample of first-time parents with a normally developing infant aged between 8 and 13 months (*N* = 153). PCA and multigroup CFA were used to investigate factorial invariance.

### Methods

#### Participants and procedures

In Study 2, participants were recruited by undergraduate students of KU Leuven, Belgium, in return for course credits in a course on research interviewing. The study was conducted between November 2009 and December 2010 as part of a broader longitudinal study, and was approved by the Ethics Committee of KU Leuven, Belgium. Participation was entirely voluntary and full anonymity was guaranteed. Inclusion criteria for parents to be eligible were (a) Dutch-speaking, (b) being in a heterosexual partner relationship so as to have one parent of each gender, and (c) being a first-time parent of a healthy biological child between the ages of 8 and 13 months. Couples who agreed to participate were visited in their homes by the students and written informed consent was obtained by both parents.

Ninety-two couples participated in the study, 84 of whom met inclusion criteria (91.30%). Of the 168 eligible parents, 153 returned the completed questionnaire booklet at Time 1 (response rate = 91.07%), including 76 mothers (49.7%) and 77 fathers (50.3%). Table 5 provides demographic features of the participants. Mothers and fathers differed significantly in age (*t*[134.62] = -3.57, *p* < .001), working status (*χ*^2^[4] = 12.64, *p* < .05), and working hours (*t*[137] = -3.07, *p* < .01). The 76 infants in this study were 45 girls (59.2%) and 31 boys (40.8%). Their mean age was 10.11 months (*SD* = 1.24; range 8–13). All infants had Belgian nationality.

**Table 5:** Demographic features of mothers and fathers in Study 2.

Parameter	Mothers (*N* = 76)	Fathers (*N* = 77)
Age (years)^a^	29.31 (3.00)	31.48 (4.39)
Belgian nationality (%)	97.4	100
Work (hours per week)^a^	34.52 (7.68)	41.71 (17.61)
Work (days per week)^a^	4.78 (0.70)	4.81 (1.40)
Educational level (%)		
Primary education	0.00	1.30
Secondary education	17.10	26.00
Higher education (3 years)	43.40	42.90
Higher education (> 3 years)	39.50	42.90
Working outside home (%)	96.10	94.80

^a^ Mean (*SD*)

#### Measures

As in Study 1, all participants completed the PRFQ, the KKL [74], and the ECR-R [75]. Estimates of internal consistency in this study were α = .79, .85, and .85 for symptoms, attachment avoidance, and attachment anxiety, respectively. In addition, participants completed the Dutch version (Nijmeegse Ouderlijke Stress Index) [88] of the Parenting Stress Index (PSI) [89], as reflective functioning has been suggested to be a key factor in buffering stress and fostering resilience in the face of stress and adversity [67, 90]. Hence, high levels of PRF might be an important buffer for stress associated with parenting specifically. In this study, we used only the following subscales, from the parent domain, to minimize participant burden: Competence (13 items; e.g., “I can’t make a decision without help”), Role Restriction (seven items; e.g., “I feel restricted by my obligations as a parent”), Social Isolation (six items; e.g., “I am less interested in other people than before”), and Marital Relationship (seven items; e.g. “Raising a child has led to more relational conflicts than expected”). Respondents are asked to rate items of the PSI on a Likert-type scale ranging from 1 (strongly disagree) to 6 (strongly agree). We also calculated a total parenting stress score by averaging the scores on the subscales. Cronbach’s alphas in this study were α = .83, .81, .60, .72, and .91 for Competence, Role Restriction, Social Isolation, Marital Relationship, and Total Parenting Stress, respectively.

#### Data analysis

Multigroup CFA with maximum likelihood estimation was used to investigate the factorial invariance of the PRFQ in mothers and fathers, following recommendations formulated by Bollen [91], Byrne [92], and Kline [93]. This multigroup comparison compared a fully unconstrained model (Model 1); a measurement weights model (Model 2) fixing the factor loadings; a measurement intercepts model (Model 3), which fixed the factor loadings and intercepts; a structural covariances model (Model 4), which also fixed the variance of the factors across both mothers and fathers; and a measurement residuals model (Model 5), which also fixed the covariances and variances of the errors. In order to compare these five models, *χ*^*2*^-difference tests were used.

### Results

#### Multigroup CFA

**Fig 2:**
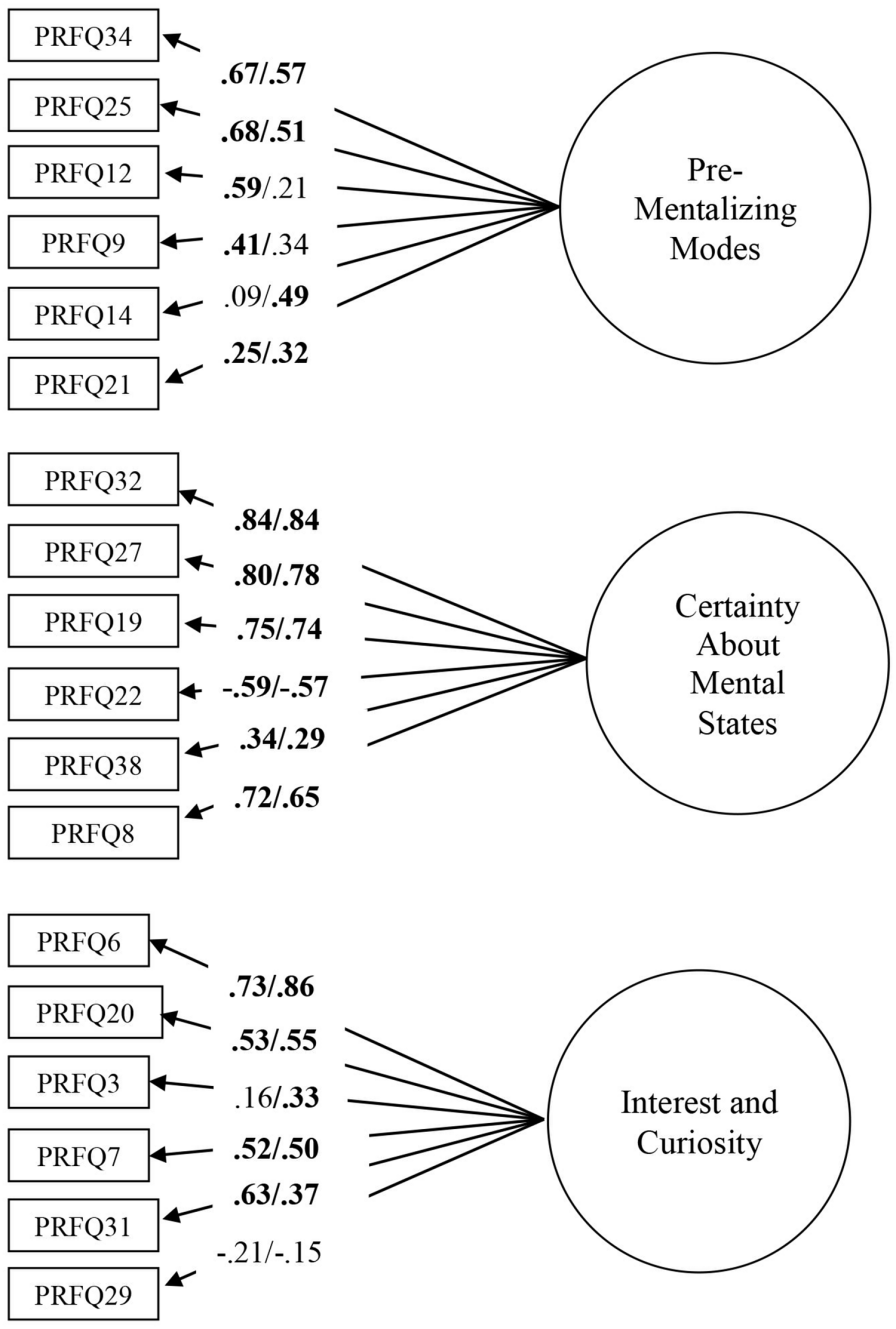
Multigroup CFA with factor loadings for fathers (left) and mothers (right) in Study 2. Residuals and correlations between residuals are omitted for clarity of presentation. Rectangles indicate measured variables and circles represent latent constructs. Standardized maximum likelihood parameters are used. Bold estimates are statistically significant.

The initial unconstrained model already had a relatively good fit: χ^2^ = 327.75, df = 258.07, *p* < .001, χ^2^/df = 1.27; RMSEA = .04 (CI .03–.06); CFI = .88, NNFI = .86. In contrast to Study 1, the correlation between CMS and IC was not significant, and hence was dropped in further models. Adding error covariances suggested by AMOS led to an excellent fit: χ^2^ = 286.83, df = 113, *p* < .001, χ^2^/df = 1.13; RMSEA = .03 (CI .00–.05); CFI = .94, NNFI = .93, suggesting factorial invariance across mothers and fathers. All subsequent models led to a significantly worse fit (measurement weights model: Δχ^2^ = 45.69, *p* < .01; measurement intercepts model Δχ^2^ = 79.61, *p* < .001; structural covariances model Δχ^2^ = 87.13, *p* < .001; measurement residuals model Δχ^2^ = 209.77, *p* < .001), suggesting that an unconstrained model fitted the data best (see Fig 2). As shown in Fig 2, for some items, item loadings for mothers and fathers differed. Moreover, some items had significant loadings in mothers but not in fathers, and vice versa. Yet, there were no mean-level differences between fathers and mothers on PM (*M* = 1.7, *SD* = .58 vs. *M* = 1.61, *SD* = .53, *t*[151] = 1.07, *ns*). However, mothers had significantly higher scores than fathers on CMS (*M* = 3.92, *SD* = 1.00 vs. *M* = 3.33, *SD* = 1.13, *t*[151] = 3.37, *p* < .001) and IC (*M* = 6.05, *SD* = .58 vs. *M* = 5.76, *SD* = .81, *t*[151] = 2.54, *p* < .001).

#### Relationships with demographic features, symptomatic distress, attachment, and parenting stress

With regard to demographic features, there were even fewer significant correlations between the PRFQ scales and these features compared with Study 1 (Table 6). As observed among the mothers in Study 1, mothers’ PM was significantly negatively related to level of education in Study 2 (*r* = -.27, *p* < .05). PM was also positively correlated with working hours (*r* = .28, *p* < .05). In fathers, however, there were no correlations between PM and demographic features. There were no significant correlations between CMS and IC and demographic features, with two small exceptions. In mothers, there was a small trend for CMS to correlate positively with child age (*r* = .20, *p* < .10), perhaps reflecting increasing knowledge about the child’s internal mental states and thus increased certainty about them. For fathers, IC was positively correlated with the duration of the partner relationship (*r* = .24, *p* < .05), perhaps reflecting fathers’ growing interest in the mental states of their child as they are in a partner relationship for longer.

**Table 6:** Correlations among the PRFQ subscales and demographic features in Study 2 for fathers (F) and mothers (M).

	Age of Parent		Parent Level of Education		Working Hours		Working Days		Duration of Relationship		Duration of Living Together		Age of Child	
	F	M	F	M	F	M	F	M	F	M	F	M	F	M
Pre-Mentalizing Modes	.08	-.14	.08	-.27*	-.03	.28*	-.18	.10	-.06	-.11	-.04	-.10	-.11	.11
Certainty about Mental States	-.04	.05	.06	.07	-.12	-.14	.05	-.04	-.04	.11	-.07	.08	-.08	.20^†^
Interest and Curiosity	-.14	.04	-.16	-.14	.01	-.09	.19	-.05	.24*	.06	.05	-.06	-.05	.09

^†^ *p* < .10 (2-tailed);

* *p* < .05 (2-tailed),

** *p* < .01 (2-tailed).

As in Study 1, in mothers, PM was positively associated with both attachment avoidance and attachment anxiety, while there was a trend for PM to correlate with symptomatic distress (Table 7). In fathers, however, there were no relationships between PM, attachment, and symptomatic distress. As in Study 1, there were no significant relationships between CMS, IC, attachment and symptomatic distress in either mothers or fathers; there was only a small trend for IC to correlate with symptomatic distress.

**Table 7:** Correlations among the PRFQ subscales, symptomatic distress, and maternal attachment in Study 2 for fathers (F) and mothers (M).

PRFQ subscales	Attachment Avoidance		Attachment Anxiety		Symptomatic Distress	
	F	M	F	M	F	M
Pre-Mentalizing Modes	.07	.23*	.12	.40**	.12	.21^†^
Certainty about Mental States	.05	-.10	.03	.01	.04	.11
Interest and Curiosity	-.09	-.09	.01	.05	.17	.20^†^

^†^ *p* < .10 (2-tailed);

* *p* < .05 (2-tailed),

** *p* < .01 (2-tailed).

Finally, for parenting stress, correlations for mothers and fathers were very similar, and thus we display correlations for mothers and fathers combined (Table 8). PM was significantly and positively correlated with total parenting stress and with all of the subscales, including role restriction, social isolation, problems in the partner relationship, and parental competence. In contrast, neither CMS nor IC was related to parenting stress.

**Table 8:** Correlations among the PRFQ subscales and parenting stress in Study 2 for fathers and mothers combined.

	Role Restriction	Social Isolation	Marital Relationship	Competence	Total
Pre-Mentalizing Modes	.25**	.23**	.18*	.34**	.29**
Certainty about Mental States	-.03	-.08	.01	-.13	-.06
Interest and Curiosity	.04	.06	-.02	-.04	.01

* *p* < .05 (two-tailed),

** *p* < .01 (two-tailed).

### Discussion

Results of Study 2 replicated the three-factor structure identified in the socially diverse sample of Study 1 in a more homogeneous sample of normally developing children and their parents. This study also provided preliminary evidence for the factorial invariance of the three-factor structure across mothers and fathers. However, there were some differences in the factor loadings between mothers and fathers, as well as in mean levels of PRF which need further research. Finally, there were also some differences in correlates with demographic features, attachment, and symptomatic distress. It may be that differences between maternal and paternal allocation of attention to their infant’s needs reflect in part changes secondary to the effects of pregnancy and the intensity of maternal caregiving, similar to what Winnicott [94] termed “primary maternal preoccupation”, which is typically more pronounced in mothers, at least in Western cultures [38, 95]. This shift in attentional allocation may facilitate more focus on the infant’s behavior and associated mental states in mothers [96]. Furthermore, differences between mothers’ and fathers’ oxytocin levels may also influence attention to the infant’s needs. Indeed, oxytocin has been shown to be important in both the initiation and maintenance of maternal behavior [97, 98], as well as affiliative relationships more generally [97, 99]. Yet, clearly, further research in larger samples is needed to investigate possible differences between maternal and paternal mentalizing.

As might be expected in this more homogeneous and well-functioning group of parents of normally developing children, relationships between the PRFQ, demographic features, symptomatic distress, and attachment were more modest than those identified in Study 1. However, there were clear relationships with parenting stress, congruent with the suggested buffering effect of PRF in both mothers and fathers that has also been found in a study using the RFS [90].

As for Study 1, the main limitation of Study 2 is that it is based on self-report questionnaires. Further replication of findings from this study is therefore needed with interview and observer-based measures. Study 3 therefore focuses on the relationship between the PRFQ and attachment behavior as assessed with the SSP, an experimental procedure that yields attachment categorizations based on coding by external observers of behavioral responses in a separation and reunion paradigm.

## Study 3

Study 3 investigated a key tenet of theories focusing on the role of PRF in child development—that this capacity fosters the development of secure infant attachment [28, 45]. Specifically, a family climate characterized by the absence of marked impairments in mentalizing and a genuine interest and curiosity in mental states is expected to be conducive to the development of secure attachment. Such a climate would provide an ideal context for the development of feelings of autonomy and effortful control, the capacity for affect regulation, and secure base feelings [46]. Furthermore, caregivers with infants with secure attachment are expected to “know” their infant’s mind relatively well, while at the same time recognizing that they can never be fully certain about their child’s wishes, thoughts, and feelings, thus recognizing the opacity of mental states [63]. With regard to the PRFQ, we therefore expected PM to be negatively associated with infant secure attachment, whereas we expected both IC and CMS to be positively associated with secure attachment. However, given the “loose coupling” of attachment and PRF, we expected high levels of PM in particular to be associated with infant insecure attachment, as the presence of a tendency to make malevolent attributions with regard to one’s child is thought to undermine the development of secure attachment [69, 100].

Study 3 addressed these predictions using data from a study on the relationship between features of parenting and the development of infant attachment as assessed by the SSP in a sample of 136 community mothers and their infants. In line with key assumptions of theories concerning the role of PRF, we expected all three PRFQ scales to predict infant attachment security.

Because of the relatively small sample size and thus the relatively small number of insecurely attached children in this sample, analyses focusing on the relationship between the PRFQ and three-way (i.e., secure, anxious-avoidant, anxious-resistant) and four-way (i.e., secure, anxious-avoidant, anxious-resistant, and disorganized) attachment classifications (Table 9) were considered to be exploratory. However, we expected PM to be associated with all insecure attachment styles, given the proposed undermining effect of PM on the formation of infant attachment.

**Table 9:** Three-way and four-way attachment classification in the SSP.

Attachment Classification	Distinguishing Features in the SSP
Secure (B)	The infant is visibly upset by separation from their caregiver, but can be relatively easily comforted upon return of the caregiver
Anxious-avoidant (A)	The infant is apparently less anxious and distressed by separation from their caregiver, and may not seek proximity with the caregiver following the separation.
Anxious-resistant (C)	The infant shows very limited exploration and play during separation from their caregiver and tends to be highly distressed by this separation. The infant is difficult to reassure after reunion with the caregiver.
Disorganized (D)^a^	The infant shows markedly inconsistent responses to separation, such as freezing and head-banging.

^a^ Included only in the four-way classification.

We expected that, relative to mothers of securely attached infants, mothers of infants with anxious-avoidant attachment would be characterized by a combination of higher levels of PM, and lower levels of IC and CMS (reflecting deactivation of attachment relationships). Anxious-avoidantly attached children typically have had experiences where their emotional arousal was not re-stabilized by the caregiver, or where they were over-aroused by intrusive parenting. As a result, they tend to over-regulate their affect and defensively inhibit distress. Hence, attachment figures of anxious-avoidant children can be hypothesized to have higher levels of PM, attributing improbable mental states to their child (e.g., “I expect my child to be obedient at all times, so if he/she is not obedient, this is because he/she wants to annoy me”). Furthermore, these attachment figures can be expected to have lower levels of IC because they are less interested in—or even tend to completely downplay the importance of—emotional experiences (e.g., “She is too young to have any feelings”). This can also be expected to lead to lower CMS relative to attachment figures of securely attached children, (e.g., “I would not know what she is feeling, I find that a strange question, she is still so young, too young to have any feelings”).

For mothers of infants with anxious-resistant attachment, we expected a combination of higher PM, lower levels of IC, and higher levels of CMS (reflecting hyperactivation of the attachment system and potentially intrusive mentalizing) relative to mothers with securely attached infants. Anxious-resistantly attached children are characterized by under-regulation of affect, that is, they typically exaggerate their expression of distress in an effort to elicit a response from what they perceive to be an unresponsive caregiver. Attachment figures of anxious-resistantly attached infants are often confused, angry or fearful in relation to their own attachment figures, and their attachment relationship with their infant tends to be heavily influenced by conflicted feelings relating to their own attachment history [34, 46], leading to a pattern of enmeshment. Hence, relative to attachment figures of securely attached infants, these caregivers can be expected to have higher levels of CMS, as they tend to confuse their own emotional experiences with those of their infant (e.g. “I always know what my child is feeling”). As a consequence, they can also be expected to show lower levels of genuine interest and curiosity (i.e., IC) in their infants’ mental states compared with caregivers of securely attached children. They may also attribute malevolent intentions to their infant, and thus show higher levels of PM, particularly when highly aroused, as they may fail to understand why their child acts or feels differently than, they expected he/she would act or feel.

Mothers of infants with disorganized attachment were expected to have higher levels of PM and lower levels of IC. These infants typically show markedly inconsistent responses to stress and arousal, such as freezing and head-banging [101]. Research suggests that caregivers of such infants are perceived as a source of both fear and reassurance, so that the arousal of the infant’s attachment system produces strong conflicting motivations [102]. Mothers of infants with disorganized attachment have very little genuine interest in their infant’s mind (low IC), but at the same time make quite improbable and often hostile assumptions about their infant’s mind (high PM). For CMS, predictions are less clear, as there are two groups of infants with disorganized attachment based on their primary attachment classification [101]. For those infants with disorganized attachment with a primary anxious-avoidant pattern, as noted above, their mothers may be characterized by relatively low levels of CMS, whereas for disorganized infants with a primary anxious-resistant pattern, mothers may have high levels of CMS relative to mothers of infants with secure attachment.

### Methods

#### Participants and procedures

Participants were mothers and their infants who were observed at 10 months of age at the Anna Freud National Centre for Children and Families (London, UK) BabyLab in the context of a broader study on the relationship between the quality of parenting and the security of the infant—parent attachment. During this visit, mothers were asked to complete a small battery of questionnaires and two experimental computerized tasks assessing implicit values about parenting. Two months later, mothers were asked to return to the BabyLab with their infants in order to assess the infant’s attachment security using the SSP. In total, 224 mother—infant dyads (46.8% boys) were recruited from the local community, using posters and flyers in children’s centers and baby clinics. As the PRFQ was added to the battery at a later date, only 136 mothers and their infants were included in Study 3. There were no differences, however, in any demographic features between mothers in the original sample and mothers who also completed the PRFQ. Demographic features of mothers and infants in Study 3 are summarized in Table 10. Most mothers were Caucasian and had attained higher education, and about half of the infants were female. This study was approved by University College London Research Ethics Committee.

**Table 10:** Demographic features of participants in Study 3.

Mean maternal age in years (*SD*)	34.24 (3.58)
Annual household income % <£30,000	19.1
Marital status, % married or living with partner	89.7
Education	
% Secondary education	10.3
% Further education	16.9
% Higher education	72.8
Mother ethnicity	
% Caucasian	88.1
% Other	11.6
Baby gender	
% (*n*) Male	46.3 (63)
% (*n*) Female	53.7 (73)

#### Measures

The SSP [46] is a widely used and well-validated measure of infant attachment [103]. It consists of eight 3-minute episodes during which the mother leaves (separation episodes) and rejoins (reunion episodes) the infant twice. Coding was based on videotaped SSPs by a trained coder for the three organized attachment classifications (secure, anxious-resistant-insecure, and anxious-avoidant-insecure) using the criteria of Ainsworth et al. [46], and attachment disorganization using the criteria of Main and Solomon [101]. All children classified as disorganized also received an alternate attachment classification (secure, anxious-resistant, and anxious-avoidant), allowing us to conduct either a four-way analysis (secure, anxious-resistant, anxious-avoidant, and disorganized) or a three-way analysis (secure, anxious-resistant, and anxious-avoidant) when disorganized infants were assigned to their alternative classifications. In total, 95 infants (70%) were classified as secure and 41 (30%) as insecure. Three-way classification led to 103 (76%) children being classified as secure, 16 (12%) as anxious-avoidant, and 17 (12%) as anxious-resistant. Four-way classification led to16 infants being categorized as anxious-avoidant (12%), 97 (71%) as secure, 11 (8%) as anxious-resistant, and 12 (9%) as disorganized. Tapes were coded by a research assistant who had passed reliability assessments on 48 tapes (kappa = 0.78 on two-way, 0.73 for three-way, and 0.80 for disorganized).

#### Statistical analyses

Binary logistic regression analysis was used to investigate the association between mothers’ PRFQ scores and attachment security in their children, using the secure versus insecure attachment classification as the dependent variable. Multinomial logistic regression was used to investigate the relationship between the PRFQ scales and the three- and four-way attachment classifications, with secure attachment as the reference category in each set of analyses. In all analyses, odds ratios (ORs) and 95% CIs were calculated. Exploratory analyses, in line with the findings in Studies 1 and 2, demonstrated small to moderate correlations between some PRFQ subscales and certain demographic features (maternal age and level of education, number of siblings, and infant gender). Therefore, all analyses controlled for infant gender, maternal age, maternal level of education, and number of siblings.

### Results

As expected, maternal PM and IC were highly significantly related to infant security of attachment (Table 11). The odds of infants being categorized as insecure were 3.05 times higher for mothers with high levels of PM. For mothers with high levels of IC, the odds of having a child with secure attachment were 2.64 times higher. CMS, however, did not predict infant attachment security.

**Table 11:** The relationship between maternal parental reflective functioning and two-, three- and four-way infant attachment classification.

Attachment Classification	PRFQ Scale	Infant Attachment Categorization	*B* (*SE*)	Wald χ^2^	*df*	Odds ratio	(95% CI)
Two-way	PRFQ-PM	Insecure	1.15 (.41)	7.54**	1	3.05	(1.38, 6.76)
	PRFQ-CMS	Insecure	-.11 (.20)	.31	1	1.11	(.76, 1.63)
	PRFQ-IC	Insecure	-1.00 (.39)	6.28**	1	2.64	(1.24, 5.63)
Three-way^a^	PRFQ-PM	Avoidant (A)	1.42 (.57)	6.19**	1	4.14	(1.35, 12.68)
		Anxious-Resistant (C)	1.33 (.54)	6.06**	1	3.78	(1.31, 10.87)
	PRFQ-CMS	Avoidant (A)	-.53 (.31)	2.89^†^	1	1.69	(.92, 3.13)
		Anxious-Resistant (C)	.33 (.276)	1.45	1	.72	(.42, 1.23)
	PRFQ-IC	Avoidant (A)	-.64 (.48)	1.74	1	1.89	(.73, 4.90)
		Anxious-Resistant (C)	-1.14 (.50)	5.26*	1	3.14	(1.18, 8.33)
Four-way^a^	PRFQ-PM	Avoidant (A)	1.38 (.57)	5.79**	1	3.96	(1.29, 12.12)
		Anxious-Resistant (C)	1.33(.63)	4.47*	1	3.78	(1.10, 12.97)
		Disorganized (D)	.09 (.71)	.02	1	1.09	(.27, 4.44)
	PRFQ-CMS	Avoidant (A)	-.56 (.32)	3.14^†^	1	1.75	(.94, 3.25)
		Anxious-Resistant (C)	.55 (.35)	2.39	1	.58	(.29, 1.16)
		Disorganized (D)	-.29 (.39)	.54	1	1.33	(.62, 2.84)
	PRFQ-IC	Avoidant (A)	-.73 (.50)	2.11	1	2.07	(.78, 5.50)
		Anxious-Resistant (C)	-2.26 (.75)	9.00**	1	9.52	(2.19, 41.67)
		Disorganized (D)	-.27 (.65)	.18	1	1.31	(4.70, 2.84)

^†^ *p* < .10,

* *p* < .05,

** *p* < .01

^a^ Odds ratios refer to index category Secure Attachment,

Exploratory analyses focusing on the three- and four-way attachment classifications showed that PM was highly significantly associated with both the anxious-avoidant and anxious-resistant pattern in both the three- and four-way classifications, with ORs ranging from 4.47 to 6.19, respectively. As expected, there was a trend for CMS to be negatively related to the anxious-avoidant pattern in infants in both the three- and four-way classifications (OR = 1.69 and 1.75, respectively), but none of these trends reached significance (Wald χ^2^ = 2.89 and 3.14, *p* = .09 and *p* = .08, respectively). By contrast, there was a slight trend for CMS to be positively related to the anxious-resistant pattern in both the three- and four-way classifications (Wald χ^2^ = 1.45 and 2.39, *p* = .23 and *p* = .12, respectively), suggesting that these mothers tended to have higher levels of CMS, whereas mothers with anxious-avoidant infants tended to have lower levels of CMS, relative to mothers with infants categorized as secure.

In line with predictions, IC was highly significantly related to the anxious-resistant attachment pattern in both the three- and four-way classifications, with ORs ranging from 5.26 to 9.00, respectively. The negative *B*s indicate that, relative to mothers of infants with secure attachment, these mothers had significantly lower levels of IC, as predicted. A similar trend was observed for anxious-avoidant attachment, but these trends failed to reach significance in analyses using either the three- or four-way attachment classification (*p* = .19 and *p* = .15, respectively).

Finally, contrary to expectations, none of the PRFQ scales was associated with disorganized attachment.

### Discussion

Results of Study 3 indicate that both PM and IC are related to infant attachment security. These findings provide further support for the validity of the PRFQ, as they confirm one of the key predictions of extant theories of PRF, namely that the capacity of parents to envision the mind of their child is related to security of attachment in their infants. The fact that infant attachment classification was assessed in the SSP, and not based on maternal self-report, lends particular credibility to these findings. Specifically, the odds of having a securely attached infant were two to three times higher for mothers with high levels of IC and low PM, respectively. It is, of course, difficult to draw any causal conclusions based on these findings, as there were only 2 months between the assessment of PRF and the assessment of infant attachment status. However, these findings are at least consistent with the notion that genuine interest and curiosity in the mental states of one’s infant, and the relative absence of pre-mentalizing modes of reflecting about the mental interior of one’s infant, is conducive to the development of attachment security in the infant. These analyses are also congruent with earlier studies that found an association between PRF and infant attachment security [30, 43, 45]. This is the first study, however, to provide a more fine-grained analysis of these associations, suggesting that both positive (i.e., interest and curiosity) and negative (pre-mentalizing modes of thinking about one’s infant) features of PRF are associated with infant attachment status.

Exploratory analyses using the three- and four-way classifications provided important leads for further research on the relationship between dimensions of PRF and specific attachment styles. Specifically, whereas PM was highly associated with both anxious-avoidant and anxious-resistant attachment, with ORs around 4, relationships for IC and CMS were more complex. These analyses also potentially shed light on the apparent absence of a relationship between CMS and infant attachment security, suggesting that this subscale might be more pertinent to the development of infant attachment *insecurity*. Findings from this study suggest, as expected, that infant anxious-avoidant attachment seems to be associated with a combination of high maternal PM and low CMS and IC relative to mothers with securely attached children. This is congruent with the notion that mothers of these infants tend to be dismissive with regard to attachment, reflecting deactivation of the attachment system and hypomentalizing. Infant anxious-resistant attachment, in turn, tended to be associated with a combination of high maternal PM and CMS and low IC, suggestive of a pattern of intrusive mentalizing that is typical of mothers showing anxious-preoccupied or disorganized attachment. Taken together, these findings suggest that a combination of relatively high levels of maternal PM and low levels of CMS and IC may be typical of dismissive mothers, increasing the probability that their infants will develop a similar dismissing (i.e., anxious-avoidant) strategy. A combination of relatively high levels of PM and CMS and low levels of IC may be characteristic of mothers with preoccupied attachment, and their entanglement with their own attachment history may lead to unjustified assumptions about their infants’ states of mind. In turn, this may lead to hypersensitivity to mental states in their infants because of these experiences of unmarked mirroring, further compounded by their mothers’ tendency for intrusive mentalizing (“knowing” what their child feels, thinks, or wants). As a result, their infants may tend to develop a similar pattern of preoccupation with attachment relationships (i.e., anxious-resistant) because of these repeated experiences of unmarked mirroring and intrusiveness. Findings from Study 1 provide further support for these assumptions, as CMS was indeed related to intrusiveness as assessed with the EA-SR scales. Further prospective research in large samples is needed, as several of these trends failed to reach significance and causality remains to be determined.

Finally, none of the PRFQ scales was associated with disorganized attachment status. This may be due to the small sample size (with only 12 infants being categorized as disorganized) and the resulting lack of statistical power, or limitations of the PRFQ to tap into the features of disorganized attachment. Interviews such as the PDI [35] and observation-based measures such as AMBIANCE [102] may be superior in tapping into the serious distortions in maternal mentalizing that may be related to attachment disorganization. Further research is clearly needed here. The absence of any relationship between the IC and CMS subscales and disorganized attachment status might be less surprising, as disorganized infants are a heterogeneous group, and these infants’ primary attachment organization might influence relationships with the IC and CMS subscales, as suggested above. Unfortunately, the small sample size precluded analyses based on the disorganized children’s primary attachment classification. Future research in more at-risk and clinical samples is therefore needed to further explore the relationship between the PRFQ and attachment disorganization.

## General discussion and conclusions

This paper reports on the development and preliminary validation of the PRFQ, a brief, multidimensional measure of PRF or mentalizing. Results obtained using both exploratory factor analysis and CFA provide evidence for three theoretically and clinically meaningful subscales; the results were replicated in two samples and were invariant across mothers and fathers. However, there was also evidence for differences between mothers and fathers in terms of PRF, which seem to be in line with extant developmental theories concerning gender differences in the transition to parenthood. Clearly, however, further research is needed in this context as there were some differences in the item loadings between mothers and fathers, and thus the meaning of items might differ between mothers and fathers. The subscales had good internal consistency, and were unrelated or only modestly related to demographic features and symptomatic distress. Moreover, the subscales were generally related in theoretically expected ways to adult attachment dimensions, emotional availability, parenting stress, and infant attachment security, consistent with a recent study reporting similar findings [104]. Overall, relationships were strongest for PM, which from a theoretical perspective can be expected, given the particularly detrimental effect of serious impairments in mentalizing about one’s infant. Results for IC and CMS were more differentiated; findings from the three studies reported in this paper suggest that average levels of both IC and CMS may be most adaptive, whereas either low or very high levels may be more maladaptive. For instance, although both IC and CMS were associated with emotional availability and IC was associated with infant attachment security, both IC and CMS were also associated with maternal intrusiveness; low levels of IC were associated with anxious-resistant and anxious-avoidant infant attachment status; and high levels of CMS tended to be associated with anxious-resistant infant attachment status, suggestive of a pattern of hypermentalizing [51].

Further research concerning the psychometric features of the PRFQ is indicated, as the results of the present studies should be seen as only preliminary and should be interpreted in the context of several limitations. First, Studies 1 and 2 were solely based on maternal self-report and were cross-sectional. Furthermore, although Study 1 included a socially diverse sample of mothers, more research in at-risk and clinical samples is needed. Further, Study 3 found that the PRFQ predicted infant attachment status in the SSP. These findings, together with two other studies showing that the PRFQ predicted parental distress tolerance in mothers in a simulated baby paradigm [64, 65], and a study reporting that the PRFQ was related to neural responses to infant face and cry perception using event-related potentials (ERPs) [66], provide some evidence that results cannot be explained solely on the basis of shared method variance. Second, the PRFQ can never yield the clinically rich, detailed, and idiosyncratic information that can be obtained with interview-based measures of PRF, such as reflective functioning scored on the AAI and PDI. The PRFQ was developed as only a brief screening tool that can be used in studies with large sample sizes. We therefore recommend that future studies use a combination of the PRFQ, possibly as an initial screening tool, and more detailed interview and/or observer-based measures to characterize a sample in greater detail. Third, the PRFQ is an “off-line” measure of PRF; that is, it assesses reflective functioning retrospectively and not during a social interaction, when stress and arousal levels are typically much higher [5, 81]. Nonetheless, the finding that the PRFQ predicted infant attachment status assessed in the SSP and parental distress tolerance in a simulated baby paradigm provide at least some support for the assumption that the PRFQ taps into features of PRF. Further research concerning the relationships between various measures of PRF is definitely needed. As noted, currently more than 15 measures of PRF exist [53] for children aged 3 and younger only, and very little is known about the relationships among all these measures, their predictive value, their neurobiological correlates, and their sensitivity to capture changes as a result of interventions [53]. It is hoped that the PRFQ, as a brief screening measure that can be easily administered in large samples, may contribute to studies exploring these issues.

Finally, although two studies have provided preliminary evidence that the PRFQ subscales are sensitive to change [105, 106], further research is needed to investigate the potential of the PRFQ to measure changes in parental reflective functioning as a result of psychosocial interventions.

To summarize, the three studies reported in this paper, together with a number of other studies [64–66, 104, 107], provide preliminary evidence for the reliability and validity of the PRFQ as a brief multidimensional measure of PRF that offers a potentially valuable methodological boost in large-scale studies of parental development and individual differences among parents.
